# Systemic immune-inflammation index and systemic inflammation response index levels are associated with coronary heart disease prevalence in the asthmatic population: a cross-sectional analysis of the NHANES 2011–2018

**DOI:** 10.3389/fpubh.2025.1514016

**Published:** 2025-07-08

**Authors:** Xian Wu, Haiyang Zhang, Hanmin Liu

**Affiliations:** ^1^Key Laboratory of Birth Defects and Related Diseases of Women and Children of MOE, Division of Pediatric Pulmonology and Immunology, NHC Key Laboratory of Chronobiology, West China Second University Hospital, Sichuan University, Chengdu, China; ^2^Key Laboratory of Birth Defects and Related Diseases of Women and Children of MOE, Department of Pediatrics, West China Second University Hospital, Sichuan University, Chengdu, China

**Keywords:** coronary heart disease, systemic immune-inflammation index, systemic inflammation response index, asthma, NHANES

## Abstract

**Background:**

Earlier studies have indicated a positive correlation systemic immune-inflammatory index (SII) and systemic inflammatory response index (SIRI) levels and the development of coronary heart disease (CHD). However, the correlation between SII, SIRI levels and the incidence of CHD in patients with asthma has not been described. The purpose of the study was to research the potential correlation between the levels of SII, SIRI and the incidence of CHD in patients with asthma.

**Methods:**

We conducted a retrospective cross-sectional analysis in which data of individuals from the National Health and Nutrition Examination Survey (NHANES) between 2011 and 2018. This study included 39,156 adults. Weighted multivariable regression analysis and subgroup analyses were used to assess the independent and combined associations between CHD prevalence and SII, SIRI levels of asthmatic population.

**Results:**

Totally, 2,321 adults were included in our analysis, with 116 participants experiencing CHD and the remaining 2,205 participants being free of CHD. SII levels did not significantly correlate with any of the participants' baseline characteristics, nor did SIRI levels (*r* < 0.1). Higher levels of SII were related to increased incidence of CHD, with an OR of 1.462 (95% CI, 1.031–1.893) (*p* < 0.001). Similarly, SIRI levels had similar results, with OR of 1.268 (95% CI, 1.095–1.441) (*p* < 0.05). Positive correlations between SII, SIRI levels and the incidence of CHD were observed (*p* < 0.05). Curve fitting further illustrated a positive correlation between SII, SIRI and the incidence of CHD in participants with asthma. Threshold effect analysis showed that higher levels of SII and SIRI were associated with a higher incidence of CHD, especially when SII and SIRI levels exceeded the thresholds of 411.238 and 1.812. Stratified analyses confirmed that the associations between higher SII and SIRI and increased CHD incidence in most subgroups remained consistent.

**Conclusions:**

The incidence of CHD in asthmatic individuals was positively correlated with elevated SII and SIRI levels among US adults. SII and SIRI serve as recently emerged inflammatory markers for assessing CHD prevalence in the asthmatic population. However, in order to confirm these findings, more rigorous large-scale prospective studies are needed.

## 1 Background

Asthma is a heterogeneous disease that is essentially a chronic inflammatory airway disease. The pathomechanism of asthma involves the participation of multiple immune cells and various inflammatory mediators, leading to airway inflammation and remodeling. Globally, the prevalence and incidence of asthma have been increasing annually (1). There are roughly 339 million people diagnosed with asthma globally, and in China, the adult asthma prevalence is around 1.3%, while the prevalence in children is higher, ranging from 3% to 5% (2). The high incidence and disease burden of asthma not only impact life quality but also bring huge healthcare and economic burdens on families and society (2, 3). Treatment for asthma include inhaled corticosteroids (ICS), bronchodilators, and leukotriene receptor blocker. Novel targeted therapies, including anti-IgE monoclonal antibodies and anti-IL-5 monoclonal antibodies, are widely used in severe asthma patients (4). While the use of asthma medications can effectively control symptoms in most patients, there is still a need for ongoing evaluation of the potential risks that may result from asthma.

Recently, many studies have highlighted asthma is not merely a respiratory disease but is also closely associated with various systemic conditions, such as obesity, metabolic syndrome, chronic kidney disease, and stroke (5–8). Notably, the relationship between asthma and CHD has attracted particular attention. CHD, characterized by myocardial ischemia due to atherosclerosis of the coronary arteries, is a high-incidence and high-mortality condition and a leading cause of death globally (9). Traditionally, risk factors for CHD include hypertension, hyperlipidemia, diabetes, and smoking. However, the role and mechanisms of chronic inflammation in the development of CHD are receiving increasing attention from researchers (10). This indicates a significant interplay between these diseases. Asthma, as a chronic inflammatory disorder, may be increase the risk of CHD through multiple mechanisms. Therefore, it is essential to investigate the direct link between asthma and CHD.

Inflammation is a complex response of to harmful stimuli. Its purpose is to protect the body, eliminate the causative agents, and initiate the repair process. However, the inflammatory response is a double-edged sword, and excessive or persistent inflammation may cause organ and tissue damage that can lead to a range of diseases. In patients with asthma, inflammation is a central mechanism underlying airway pathology. Therefore, controlling airway inflammation is a core objective in the management of asthma and a primary strategy in its treatment (4). Similarly, inflammation is involved in the development of CHD. Endothelial cells in the coronary arteries, when exposed to various harmful factors such as hypertension, hyperglycemia, and smoking, can activate an inflammatory response. As a result, inflammatory cells, such as MONO and macrophages, are recruited around the endothelium, forming lipid streaks that eventually develop into atherosclerotic plaques (11). During this process, inflammatory mediators, including tumor necrosis factor-α (TNF-α), interleukin-5 (IL-5), interleukin-6 (IL-6) and histamine not only promote the activation of endothelial cells but also lead to abnormal behavior of smooth muscle cells, such as migration and proliferation, which could push to the development of cardiovascular diseases (12). The inflammatory response has similar pathophysiologic processes in asthma and CHD, indicating a potential interplay between these two conditions.

Several biomarkers are used to assess the inflammatory state, including white blood cell (WBC), neutrophils (N), C-reactive protein (CRP), IL-6, erythrocyte sedimentation rate (ESR), procalcitonin, fibrinogen, and TNF-α. However, these inflammatory indicators are primarily associated with infection and do not adequately reflect the immune status of the body. Composite biomarkers, like N/lymphocytes (L) ratio (NLR) and platelets (PLT)/L ratio (PLR), have emerged and are gaining increasing attention in clinical applications. Many researchers believe that both higher NLR and PLR are closely related to multiple chronic diseases, such as chronic kidney disease (13), diabetes (14), cardiovascular disease (15), and malignancies (16).

SII and SIRI are recently developed inflammatory biomarkers. SII is composed of N, L, and PLT (17), while SIRI consists of N, L, and monocytes (MONO) (18). Compared to the peripheral blood cell composite markers NLR and PLR, the innovative inflammatory indicators, including SII and SIRI, can better reflect the body's inflammatory and immune state, including local inflammatory responses, systemic inflammatory burden, and immune function. Consequently, they can predict the occurrence and prognosis of CHD. Due to the easy availability of SII and SIRI, the present validation is predictive in several diseases and is expected to be popularized in clinical applications.

Since SII and SIRI are effective indicators of systemic inflammation, it is crucial to investigate their relationship with the incidence of CHD in asthmatic individuals. The study sought to examine the correlation between SII, SIRI and the incidence of CHD by conducting a cross-sectional analysis. By identifying potential inflammatory biomarkers linked to CHD, our findings may inform future research on targeted interventions for high-risk populations.

## 2 Subjects and methods

### 2.1 Data and sample sources

All data for this research were collected from the NHANES database from 2011 through 2018. This study was a cross-sectional design with a multi-stage, categorical, cluster random sampling method to ensure a representative sample. The detailed sampling and data collection process has been reported in previous studies (19).

#### 2.1.1 Inclusion and exclusion criteria

**Figure 1:**
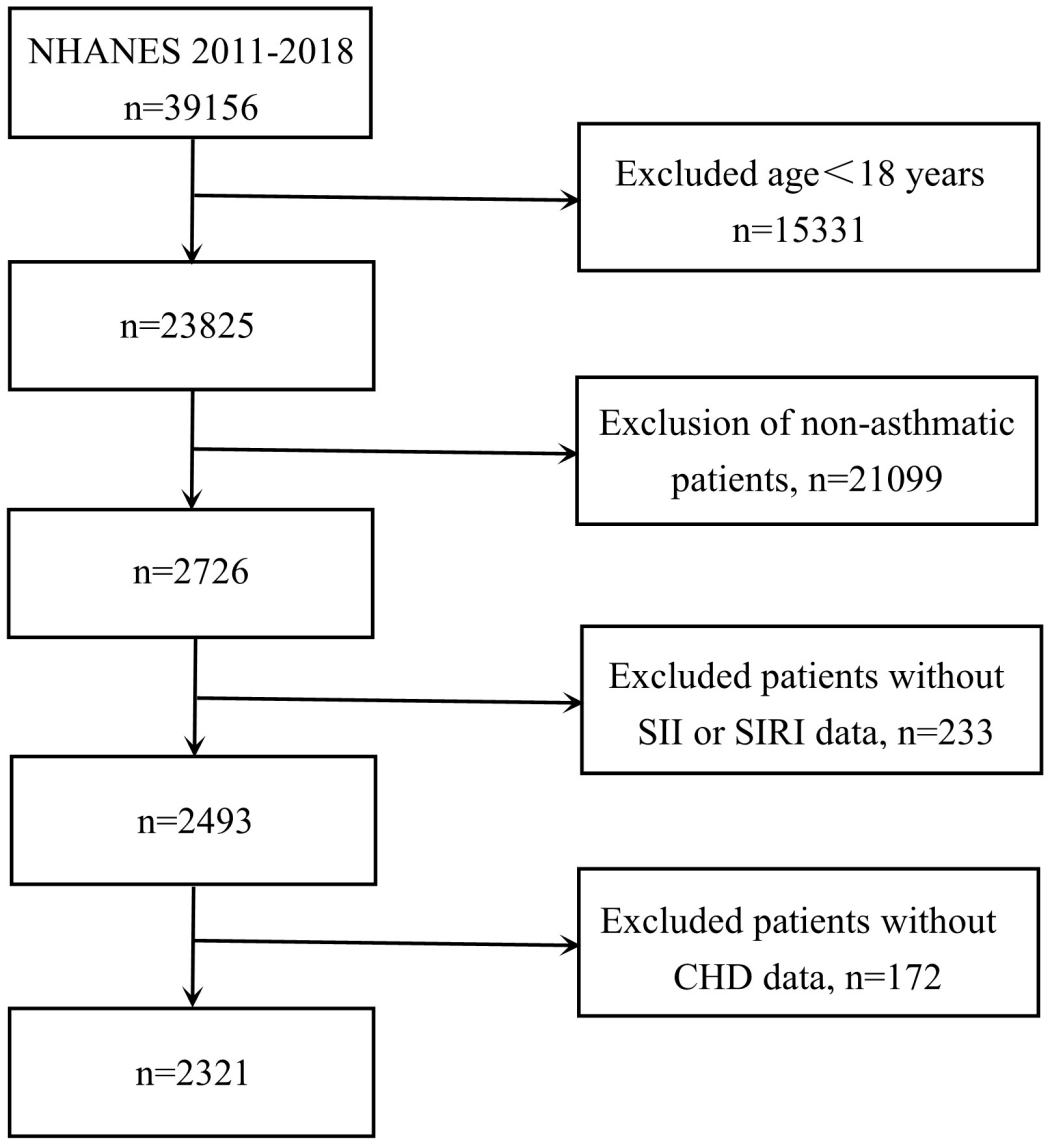
Flowchart of the sample selection from NHANES 2011–2018.

For participant selection, participants were required to meet the following criteria: (1) age ≥18 years; (2) physician-diagnosed asthma; (3) complete data on blood cell counts and CHD status. Initially, 39,156 individuals were considered. After applying our exclusion criteria, we removed those under 18 years old (*n* = 15,331), non-asthmatic participants (*n* = 21,099), cases with incomplete blood cell count data required for calculating SII and SIRI values (*n* = 233), and patients lacking CHD data (*n* = 172). The final analysis included 2,321 eligible participants (depicted in Figure 1).

### 2.2 Definition of SII and SIRI

Hematological evaluations, including complete blood counts, were performed using an automated laboratory instrument (Beckman Coulter MAXM). PLT, N, MONO, and L were measured and reported in units of × 103 cells per microliter. SII and SIRI were calculated respectively using the following formulas reported in the literature: SII = PLT × N/L, SIRI = MONO × N/L (17, 18).

### 2.3 Diagnosis of asthma and CHD

The diagnosis of asthma was derived from a self-administered questionnaire conducted by individuals when they were visited. The questionnaire included two questions: (1) “Has a doctor or other healthcare provider ever told you that you have asthma?” and (2) “Have you experienced wheezing or whistling in your chest in the past 12 months?” Participants providing positive responses to both survey items were categorized as asthmatic.

Similarly, the diagnosis of CHD was founded on self-reported medical history. Specifically, individuals were asked: “Has a doctor or other healthcare provider ever told you that you have coronary heart disease?” Based on their responses, individuals were divided into the “CHD” group and the “non-CHD” group.

### 2.4 Other variables

Other covariates mainly included sociodemographic characteristics, lifestyle behaviors, medical conditions. Sociodemographic characteristics included gender (Male, Female), age (years), race (Mexican American, Non-Hispanic White, Non-Hispanic Black, and other races), education level (<11th grade, High school graduate, College graduate or above), and BMI (kg/m^2^) (BMI was calculated by dividing weight in kilograms by height in meters squared). Lifestyle behaviors included smoking status: active smoker (>100 cigarettes/lifetime and currently smoking on certain days or every day) or non-active smoker (including never <100 cigarettes/lifetime). Medical conditions included hypertension (Yes/No), diabetes (Yes/No), cancer (Yes/No), thyroid problem (Yes/No), stroke (Yes/No), arthritis (Yes/No), high cholesterol (Yes/No). The diagnosis of hypertension was based on a definition of mean systolic blood pressure >130 mmHg or mean diastolic blood pressure >80 mmHg (20), or self-reporting of doctor's diagnosis of hypertension. The physician‘s diagnosis of diabetes mellitus was based on receiving oral hypoglycemic agents or insulin, the hemoglobin concentrations were higher than or equal to 6.5% or fasting glucose higher than or equal to 7.0 mmol/l, or random fasting glucose higher than or equal to 11.1 mmol/l or 2-h glucose tolerance glucose higher than or equal to 11.1 mmol/l or self-reported of a doctor's diagnosis of diabetes (21). The diagnosis of high cholesterol was based on a definition of high cholesterol level higher than ≥240 mg/dL (≥6.22 mmol/L), or self-reporting of doctor's diagnosis of high cholesterol level. Cancer, thyroid problem, stroke and arthritis were self-reported. All data and definitions related to these variables are publicly accessible at https://www.cdc.gov/nchs/nhanes/.

### 2.5 Statistical analysis

Continuous data are presented in mean and standard deviations (SD) format. To evaluate differences between groups categorized by tertiles of SII and SIRI, weighted *t*-tests were employed. To evaluate potential relationships between SII, SIRI and demographic parameters, we employed Spearman's rank-correlation coefficient analysis (22). A correlation coefficient (*r*) <0.1 indicates no correlation, 0.1–0.3 indicates low correlation, 0.4–0.6 indicates moderate correlation, and 0.7–1.0 indicates significant correlation.

Categorical data were presented as percentages, and weighted chi-square tests were applied to assess differences between variables. The study evaluated the link between CHD incidence and SII/SIRI through a three-step multivariate logistic regression model. Since the data came from the NHANES survey, complex sampling weights were used in the analysis, and weighted logistic regression analysis was performed in the “Multiple Regression Equations” module of Empower Stats to correct for sampling design bias.

No variables were adjusted in Model 1. Following there variables, including age, gender, and ethnicity were adjusted in Model 2. Model 3 further accounted for additional variables, including BMI, ALT, AST, smoking status, cancer, hypertension, high cholesterol, diabetes, arthritis, gout, stroke, thyroid problems, chronic bronchitis, emphysema and chronic obstructive pulmonary disease (COPD). In addition, we investigated the non-linear associations and inflection points between SII, SIRI and CHD using smoothed curve fitting and threshold effect analysis models. Threshold effect analysis was conducted using the dedicated “Threshold Effect Analysis” module in Empower Stats. By constructing a restricted cubic spline model, multiple nodes were set to allow variables to exhibit smooth non-linear trends, thereby assessing the non-linear relationship between SII and SIRI and the prevalence of coronary heart disease in the asthma population.

Subgroup analyses were carried out to measure the correlation between CHD incidence, SII and SIRI. The subgroup factors were considered predefined potential effect modifiers, and interaction terms were included to assess variations in associations across different subgroups.

To manage missing data, we employed Multiple Imputation by Chained Equations (MICE) under the missing-at-random (MAR) assumption (23). Statistical analyses were executed using R software (version 3.4.3) and supplemented with Empower Stats software (http://www.empowerstats.com).

## 3 Results

### 3.1 Baseline characteristics

Regarding inclusion criteria, we first excluded 15,331 children under 18 years of age. Next, 21,099 participants who lacked information on asthma were excluded, as well as 233 participants without LYM, N, PLT and MONO data. In addition, we analyzed CHD prevalence and excluded 172 participants without CHD information, as detailed in Figure 1. The subjects obtained had a low rate of missing covariates (all <10%), as detailed in Supplementary Table S1.

The baseline demographic data for individuals with asthma from NHANES 2011 to 2018 was displayed in Table 1. There were 2,321 participants totally included, with 41.4% (*n* = 960) being male and 58.6% (*n* = 1,361) being female. Among these participants, 116 had a history of CHD, while 2,205 did not. The CHD group participants had a significantly higher BMI and a higher prevalence of diabetes, hypertension, stroke, smoking, and high cholesterol (*p* < 0.05) compared to those in non-CHD group. According to the Spearman rank correlation analysis, there was no significant correlation between SII and SIRI levels and baseline characteristics (*r* < 0.1) (Supplementary Table S2).

**Table 1:** Baseline characteristics of individuals with asthma from NHANES 2011–2018.

**Characteristics**	**Non-CHD (*n* = 2,205)**	**CHD (*n* = 116)**	***p*-value**
**Age**	46.446 ± 17.545	66.267 ± 11.237	<0.001
**Gender**			0.002
Male	896 (40.635%)	64 (55.172%)	
Female	1,309 (59.365%)	52 (44.828%)	
**BMI**	29.627 ± 7.288	30.791 ± 8.264	<0.001
≤ 18.5	34 (1.560%)	5 (4.464%)	
>18.5, ≤ 25	385 (17.460%)	25 (%)	
>25, ≤ 30	837 (37.96%)	41 (%)	
>30	949 (43.04%)	45 (%)	
**Race**			0.412
Mexican American	204(9.252%)	5(4.310%)	
Non-Hispanic White	935(42.404%)	69(59.483%)	
Non-Hispanic Black	552(25.034%)	16(13.793%)	
Other Race	514(23.311%)	26(22.414%)	
**Education**			0.226
Less than 11th grade	442 (20.036%)	29 (25.000%)	
High school graduate	447 (20.263%)	24 (20.690%)	
College graduate or above	1,309 (59.365%)	62 (53.448%)	
**Blood count**			0.052
WBC	7.484 ± 2.396	8.188 ± 4.115	
LYM	2.209 ± 1.108	2.181 ± 3.157	<0.001
MONO	0.568 ± 0.201	0.643 ± 0.302	0.002
N	4.427 ± 1.808	5.024 ± 2.421	0.011
HGB	13.851 ± 1.523	13.460 ± 1.667	0.029
PLT	245.119 ± 62.642	223.422 ± 71.597	<0.001
SII	543.164 ± 362.652	753.497 ± 381.558	0.025
SIRI	1.292 ± 1.111	2.106 ± 2.031	<0.001
ALT	25.370 ± 13.762	31.339 ± 55.507	0.282
AST	24.834 ± 17.424	34.393 ± 127.374	0.285
**Cancer**			0.002
Yes	223 (10.113%)	25 (21.552%)	
No	1979 (89.751%)	91 (78.448%)	
**Smoke**			<0.001
Active smoker	1,034 (46.893%)	80 (68.966%)	
Non-active smoker	1,169 (53.016%)	35 (30.172%)	
**Thyroid problem**			0.055
Yes	63 (2.857%)	48 (41.379%)	
No	1908 (86.531%)	93 (80.172%)	
**Gout**			0.316
Yes	114 (5.170%)	17 (14.655%)	
No	2,090 (94.785%)	98 (84.483%)	
**Diabetes**			<0.001
Yes	397 (18.054%)	51 (43.965%)	
No	1,800 (81.855%)	65 (56.034%)	
**Hypertension**			<0.001
Yes	888 (40.382%)	91 (78.448%)	
No	1310 (59.573%)	24 (20.690%)	
**Stroke**			<0.001
Yes	102 (4.638%)	26 (22.414%)	
No	2,095 (95.271%)	90 (77.586%)	
**Arthritis**			0.427
Yes	753 (34.243%)	83 (71.552%)	
No	1442 (65.575%)	33 (28.448%)	
**High cholesterol**			<0.001
Yes	729 (33.151%)	80 (68.966%)	
No	1,457 (66.257%)	35 (30.172%)	

CHD, coronary heart disease; BMI, body mass index; WBC, white blood cell; LYM, lymphocyte; MONO, monocytes; N, neutrophil; HGB, hemoglobin; PLT, platelets; SII, systemic immune-inflammatory index; SIRI, systemic inflammatory response index; AST, aspartate transaminase; ALT, aspartate aminotransferase.

### 3.2 The association between SII and increased incidence of CHD

Across multiple models, the relationship remained consistent: Model 1 showed an OR of 2.165 (95% CI: 1.521–2.809; *p* = 0.006), Model 2 showed an OR of 2.057 (95% CI: 1.025–3.089, *p* = 0.007), and even after full adjustment in Model 3, the OR was 1.462 (95% CI: 1.031–1.893; *p* < 0.001), which indicating that the increase of each unit in SII was associated with an average 1.462-fold increase in the incidence of CHD (Table 2).

**Table 2:** Association between systemic immune-inflammation indicator (SII) and systemic inflammation response index (SIRI) levels and CHD prevalence in the asthmatic population.

**Exposure**	**Model 1 OR (95% CI) *p* value**	**Model 2 OR (95% CI) *p* value**	**Model 3 OR (95% CI) *p* value**
SII	2.165 (1.521, 2.809) 0.006	2.057 (1.025, 3.089) 0.007	1.462 (1.031, 1.893) <0.001
Tertile 1	Reference	Reference	Reference
Tertile 2	1.521 (0.792, 2.25)	1.684 (0.687, 2.681)	1.392 (1.191, 1.593)
Tertile 3	2.102 (0.951, 3.253)	2.152 (1.215, 3.089)	1.524 (1.105, 1.943)
*p* for trend	0.213	0.127	0.001
SIRI	1.469 (1.226, 1.712) <0.001	1.354 (1.024, 1.684) 0.006	1.268 (1.095, 1.441) 0.042
Tertile 1	Reference	Reference	Reference
Tertile 2	1.275 (1.026, 1.524)	1.293 (1.016, 1.570)	1.326 (1.043, 1.609)
Tertile 3	1.364 (0.891, 1.837)	1.482 (1.101, 1.863)	1.419 (1.205, 1.633)
*p* for trend	0.226	0.032	<0.001

SII and SIRI were converted from a continuous variable to a categorical variable (tertiles). Model 1: no covariates were adjusted. Model 2: adjusted for gender, age, and race. Model 3: adjusted for gender, age, race, BMI, ALT, AST, smoke, cancer, hypertension, high cholesterol, diabetes, arthritis, gout, stroke, thyroid problem, chronic bronchitis, emphysema and COPD were adjusted.

95% C1, 95% confidence interval; SII, systemic immune-inflammatory index; SIRI, systemic inflammatory response index

To validate these findings, we divided SII into three tertiles for sensitivity analysis. Compared to individuals in the lowest tertile, these in the highest part had a higher incidence of CHD (OR = 1.524; 95% CI: 1.105–1.943). Additionally, a notable increase in CHD incidence was observed in the middle tertile relative to the individuals in the lowest tertile (OR = 1.392; 95% CI: 1.191–1.593).

**Figure 2:**
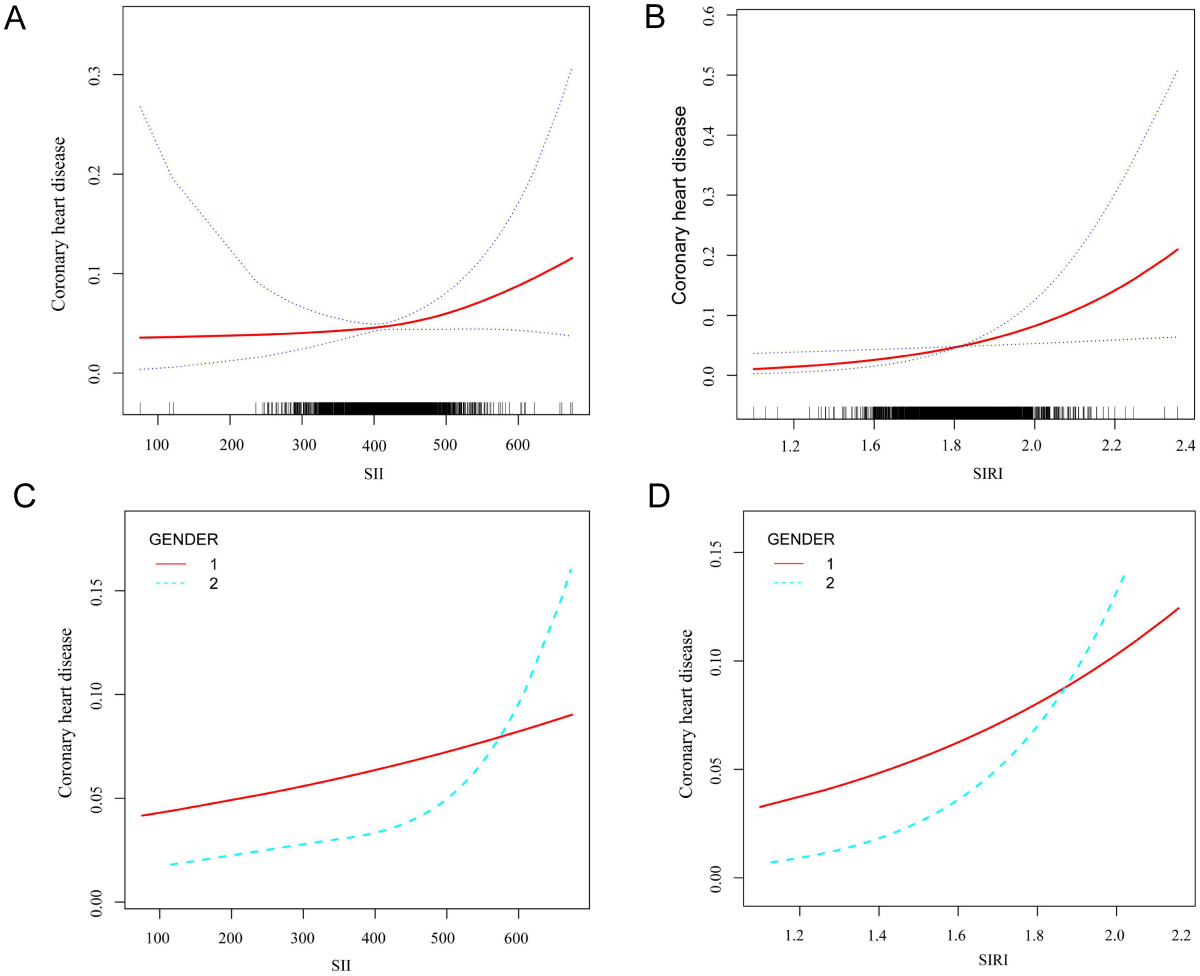
Smooth curve fitting detected the relationship between SII, SIRI and CHD. **(A)** The non-linear relationship between SII and the incidence of CHD in participants diagnosed with asthma. **(B)** Differences in CHD incidence risk between men and women at different SII levels. 1 represents men, and 2 represents women. **(C)** The non-linear relationship between SIRI and the incidence of CHD in participants diagnosed with asthma. **(D)** Differences in CHD incidence risk between men and women at different SIRI levels. 1 represents men, and 2 represents women.

Figure 2 illustrated the non-linear relationship SII and the incidence of CHD in participants diagnosed with asthma, adjusting for variables such as BMI, ALT, AST, smoking status, cancer, hypertension, high cholesterol, diabetes, arthritis, gout, stroke, and thyroid problems, chronic bronchitis, emphysema and COPD (Figure 2A). Analysis of the threshold effect showed a significant rise in CHD incidence among asthma patients with higher SII levels, especially when SII level went beyond the threshold of 411.238 (Figure 2A). Before reaching the inflection point, the incidence of CHD rose at a relatively slow pace as the SII increased. Conversely, after crossing the inflection point, the incidence of CHD escalated markedly with further increases in the SII (Supplementary Table S3). Within the low SII range, the prevalence of coronary heart disease in men remains higher than in women. Within the high SII range, the prevalence of coronary heart disease in women rapidly increases and exceeds that in men (Figure 2B).

### 3.3 The relation between SIRI and increased incidence of CHD

Our results demonstrated a positive association between increased SIRI and the incidence of CHD among asthmatic individuals. Across multiple models, the relationship remained consistent: Model 1 showed an OR of 1.469 (95% CI: 1.226–1.712; *p* < 0.001), Model 2 showed an OR of 1.354 (95% CI: 1.024–1.684; *p* = 0.006), and even after full adjustment in Model 3, the OR was 1.268 (95% CI: 1.095–1.441; *p* = 0.042), which demonstrating that the increase of each unit in SIRI was associated with an average 1.268-fold increase in the incidence of CHD (Table 2).

Compared to individual in the lowest tertile, those in the highest part demonstrated a statistically significant increase in CHD incidence (OR = 1.419; 95% CI: 1.205–1.633). The middle SIRI tertile also showed a trend toward higher CHD incidence compared to the lowest tertile (OR = 1.326; 95% CI: 1.043–1.609) (shown in Table 2).

Next, the non-linear relationship was examined between SIRI and CHD incidence in asthmatic individuals, adjusting for variables such as BMI, ALT, AST, smoking status, cancer, hypertension, high cholesterol, diabetes, arthritis, gout, stroke, and thyroid problems, chronic bronchitis, emphysema and COPD. Our analysis revealed a curved, saturating pattern indicative of non-linearity (*p* for non-linearity <0.05), as depicted in Figure 2C. Notably, we identified 1.812 as the turning point in this relationship using threshold effect analysis (Supplementary Table S2), indicating that prior to the inflection point, the incidence of CHD increased relatively slowly as the SIRI increased, however, beyond the inflection point, the incidence of CHD increased significantly as the SIRI increased further (Figure 2C). Within the low range of the systemic immune-inflammatory index (SIRI), the prevalence of coronary heart disease (CHD) in men remains higher than in women. Conversely, within the high range of SIRI, the prevalence of CHD in women rapidly increases and exceeds that in men (Figure 2D).

### 3.4 Subgroup analysis

**Figure 3:**
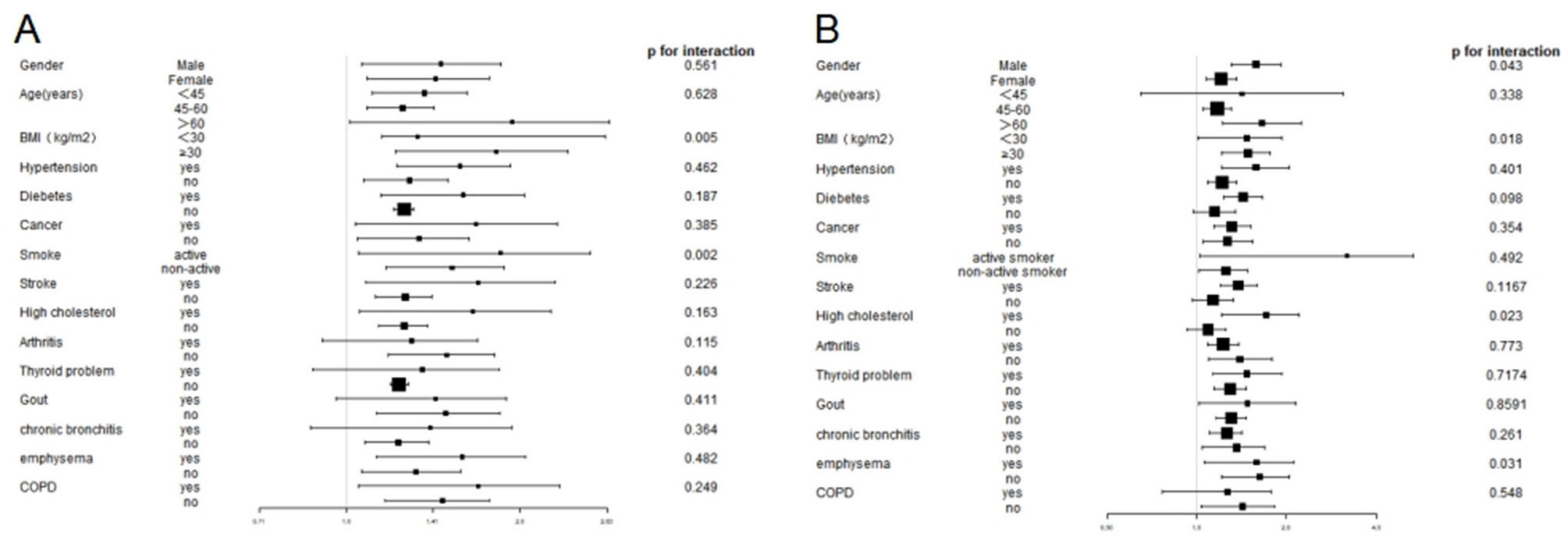
Subgroup analysis of the association between SII and SIRI levels and CHD prevalence in the asthmatic population. **(A)** Subgroup analysis of the association between SII levels and CHD prevalence. **(B)** Subgroup analysis of the association between SIRI levels and CHD prevalence.

Stratified analyses revealed that the associations between elevated SII and SIRI levels and increased CHD incidence remained consistent across various demographic and clinical characteristics in adults with asthma (Figure 3). Significant differences were observed in most subgroups, indicating that a significant positive association between SII and CHD incidence was observed. However, no substantial association was found between SII levels and the development of CHD in people with comorbidities such as with arthritis, thyroid problem, gout emphysema and chronic bronchitis. Interaction tests showed no interaction was observed in most analyses (*p* > 0.05), except for the BMI and smoking groups (*p* = 0.0013 and *p* = 0.013) (Figure 3A). Specifically, a stronger correlation between higher SII levels and CHD incidence was observed in the subgroups with BMI ≥ 28 kg/m^2^ and active smoker than in the subgroups with BMI <28 kg/m^2^ and non-active smoker. No interaction was observed in the remaining subgroups, suggesting a high degree of consistency of our findings across most of the different subgroups.

Similarly, the analysis identified notable variations across the majority of subgroups between SII levels and the development of CHD, with exceptions observed in participants with age <45 and COPD, without diabetes, stroke, and high cholesterol. The interaction analysis revealed a statistically significant difference in the gender, BMI, high cholesterol and emphysema (*p* < 0.05), whereas no other subgroups exhibited statistical significance (*p* > 0.05) (Figure 3B).

## 4 Discussion

The present study was cross-sectional and included 2,321 adult participants. We observed that individuals with higher SII, SIRI had an increased prevalence of CHD. Smoothed curve fitting showed the relationship between SII,SIRI and CHD prevalence was non-linear. Subgroup analysis and interaction tests revealed that this association was consistent in most subgroups. Higher SIRI and SII levels were positively correlated with the prevalence of CHD, indicating that SIRI and SII can be applied as important markers for predicting the likelihood of CHD in asthmatic individuals.

Several biomarkers are traditionally used to assess inflammation, including WBC, ESR, CRP, IL-6, and TNF-α. However, these are single indicators and have limitations in providing a comprehensive evaluation of the inflammatory response. SII and SIRI are recently developed composite inflammatory markers. On account of the non-invasive nature, simplicity, low cost, and ease of measurement, SII and SIRI are increasingly favored by clinicians. Compared to other single inflammatory markers, SII and SIRI provide a more comprehensive reflection of both inflammatory and immune responses.

Several studies have found patients with asthma, whether symptomatic or in remission, exhibit elevated levels of *N* in their peripheral blood (24). N significantly contribute to the development of asthma (25). Conversely, L in the peripheral blood of asthma individuals are reduced, possibly due to the redistribution of L from the bloodstream to the airway tissues, contributing to airway inflammation (26). Additionally, some researchers have found MONO‘s number in blood of asthma individuals is also increased. MONO can release various pro-inflammatory factors, exacerbating airway inflammation and contributing to airway remodeling (27, 28). Furthermore, asthma patients' PLT are also elevated, which is one of the reasons for their hypercoagulable state (29). PLT may interact with other cells in the peripheral blood to influence the body's inflammatory and immune responses (30). Multiple researches have verified the predictive capabilities of SII and SIRI. These composite inflammatory indices enhance the predictive value for various chronic diseases.

Although researchers have established a positive correlation between the levels of novel inflammatory markers SII and SIRI and the risk of CHD (31), their relationship with CHD in asthma patients remains unclear. Consequently, we aimed to explore the association between SII, SIRI and the incidence of CHD in asthma patients. Data in our study indicate that asthmatics with higher SII and SIRI are more likely to develop coronary artery disease. Epidemiological studies have indicated that CHD prevalence is significantly higher in asthma patients compared with non-asthma populations (32, 33). Some observational studies have also identified asthma as a risk factor for CHD (34, 35). However, the causal relationship between asthma and CHD remains uncertain. Several potential reasons underlie this relationship. First, asthma and CHD share common risk factors, including smoking, hyperlipidemia, obesity, air pollution, and use of inhaled bronchodilators (36, 37). The immune system in asthma patients is chronically activated, leading to immune dysregulation. This imbalance not only exacerbates airway inflammation but may also affect the systemic immune system, increasing the risk of CHD. Elevated SII and SIRI levels reflect this immune dysregulation, indicating ongoing inflammatory activity. Second, activated airway inflammatory cells in asthma patients, such as eosinophils, N, and MONO, may migrate to other organs, including the heart and blood vessels, via the bloodstream (38). These inflammatory cells continue to activate within the coronary arteries, promoting the the onset and advancement of atherosclerosis (39). Third, patients with asthma are in a chronic inflammatory state for a long time and are detected to have increased levels of several recognized inflammatory markers (40). These inflammatory cytokines can accelerate the formation and advancement of atherosclerosis, leading to increase the risk of CHD (41). These cytokines act through multiple mechanisms, including endothelial dysfunction, lipid deposition, and inflammatory cell infiltration, to hasten the atherosclerotic process (42). Oxidative stress accompanying inflammation can damage vascular endothelial cells, leading to impaired endothelial function, which is an initial indicator of atherosclerosis. Dysfunction of the endothelium further promotes lipid deposition and inflammatory cell infiltration, ultimately resulting in plaque formation and instability (42). Additionally, asthma patients frequently have metabolic syndrome, including obesity, insulin resistance, and hyperlipidemia, which are closely associated with CHD (43). Moreover, asthma patients may experience reduced physical activity and unhealthy lifestyle choices due to disease symptoms or medication use (such as long-term glucocorticoid therapy), further increasing the risk of cardiovascular disease (44, 45).

The findings of our study may have some important clinical implications. First, SII and SIRI, as simple and accessible inflammatory indicators, may be serve as tools for evaluating the risk of CHD in asthma patients, aiding in the early detection and timely management of high-risk patients. Second, monitoring changes in these inflammatory markers can help evaluate treatment efficacy and guide adjustments to therapeutic regimens, thereby improving patient outcomes. Finally, our result present new insights to the inflammatory mechanisms linking asthma and CHD, laying the groundwork for further research and the development of novel strategies for treatment. As to individuals with both asthma and CHD, a comprehensive approach to the treatment and management of both conditions should be adopted to reduce the risk of cardiovascular events.

However, there are some limitations to our study. First, our study was a cross-sectional study, so we have not yet been able to illuminate a causal correlation between SII and SIRI levels and the likelihood of developing CHD in patients with asthma. The longitudinal study is needed to verify this viewpoint in the future. Second, self-reported questionnaires were to diagnosis both asthma and CHD in this study, which may introduce recall bias and reduce diagnostic accuracy compared to medical diagnoses. Additionally, we lacked data on asthma subtypes, making it unclear how asthma phenotypes and disease severity influenced our results. Third, although we adjusted for potential confounders in our regression analyses, we could not account for other variables such as comorbid inflammatory diseases, dietary patterns, physical activity, medication use, and genetic background, which may affect the conclusions. Fourth, all data involved in this study were limited to participants in US and the results in our study can not apply to populations in other countries and of other races. What‘s more, the asthma diagnosis was based on self-reported questionnaires rather than objective pulmonary function tests. Wheezing symptoms associated with asthma may overlap with cardiac pathologies. Although we adjusted for cardiovascular risk factors including hypertension and diabetes in multivariable analyses, residual confounding from undiagnosed cardiac conditions cannot be entirely excluded. Given these limitations, more research is essential to explore the role of SII and SIRI in the prevalence of CHD among asthma patients. Such studies will help predict the likelihood of asthma patients developing CHD and provide data to support the enhancement of treatment strategies for better efficacy for patients with both asthma and CHD.

## 5 Conclusion

In asthma patients, elevated levels of SII and SIRI SIRI showed a significant association with a higher incidence of CHD, regardless of baseline characteristics or pre-existing conditions. Monitoring these inflammatory markers can enhance our ability to assess CHD risk in asthma patients and offer new perspectives for clinical management and treatment. Further validation of these findings is necessary through rigorous, large-scale prospective studies.

## Data Availability

The datasets presented in this study can be found in online repositories. The names of the repository/repositories and accession number(s) can be found below: https://www.cdc.gov/nchs/nhanes.
