# Supplementary material for: Systemic immune-inflammation index and systemic inflammation response index levels are associated with coronary heart disease prevalence in the asthmatic population: a cross-sectional analysis of the NHANES 2011–2018
Source: Front Public Health. 2025 Jul 8;13:1514016. doi: 10.3389/fpubh.2025.1514016 (PMC12279799; doi:10.3389/fpubh.2025.1514016)
Supplement: Supplementary file 1 [file Data_Sheet_1.docx]

Table S1 Missing data handling

| Item | Sample size | Missing number | Missing percentage | Handle method |
| --- | --- | --- | --- | --- |
| Age | 2321 | 0 | 0 | none |
| Gender | 2321 | 0 | 0 | none |
| BMI | 2321 | 56 | 2.40% | median replacement and MICE |
| Race | 2321 | 0 | 0 | none |
| Education | 2321 | 25 | 1.10% | most frequent category replacement and MICE |
| Blood count | 2321 | 0 | 0 | none |
| AST | 2321 | 125 | 5.40% | median replacement and MICE |
| ALT | 2321 | 125 | 5.40% | median replacement and MICE |
| Cancer | 2321 | 64 | 2.80% | most frequent category replacement and MICE |
| Smoke | 2321 | 90 | 3.90% | most frequent category replacement and MICE |
| Thyroid problem | 2321 | 142 | 6.10% | most frequent category replacement and MICE |
| Gout | 2321 | 23 | 1.00% | most frequent category replacement and MICE |
| Diebetes | 2321 | 45 | 1.90% | most frequent category replacement and MICE |
| Hypertension | 2321 | 74 | 3.20% | most frequent category replacement and MICE |
| Stroke | 2321 | 102 | 4.40% | most frequent category replacement and MICE |
| Arthritis | 2321 | 83 | 3.60% | most frequent category replacement and MICE |
| High cholesterol | 2321 | 23 | 1.00% | most frequent category replacement and MICE |

BMI: body mass index; AST: aspartate transaminase; ALT: aspartate aminotransferase. MICE: Multiple Imputation by Chained Equations

Table S2 Spearman correlation analysis of SIl, SIRI and baseline characteristies

|  |  | Correlation  (r) | P-value |  |  | Correlation  (r) | P-value |
| --- | --- | --- | --- | --- | --- | --- | --- |
| SII | Age | 0.013 | 0.045 | SIRI | Age | 0.077 | 0.249 |
|  | Gender | -0.039 | 0.004 |  | Gender | 0.082 | 0.025 |
|  | BMI | 0.047 | <0.001 |  | BMI | -0.068 | 0.003 |
|  | Race | 0.032 | <0.001 |  | Race | -0.035 | 0.452 |
|  | Education | -0.015 | 0.344 |  | Education | 0.054 | <0.001 |
|  | Blood count | | |  | Blood count | | |
|  | WBC | -0.041 | 0.031 |  | WBC | -0.071 | 0.045 |
|  | LYM | -0.028 | 0.607 |  | LYM | -0.056 | 0.209 |
|  | MONO | 0.005 | <0.001 |  | MONO | -0.089 | 0.005 |
|  | ANC | 0.038 | 0.045 |  | ANC | 0.016 | 0.057 |
|  | HGB | -0.044 | 0.511 |  | HGB | -0.027 | 0.403 |
|  | PLT | 0.023 | 0.038 |  | PLT | 0.083 | 0.039 |
|  | SII | -0.011 | 0.032 |  | SII | 0.027 | <0.001 |
|  | SIRI | 0.035 | 0.020 |  | SIRI | 0.097 | 0.398 |
|  | ALT | -0.008 | 0.423 |  | ALT | 0.049 | 0.641 |
|  | AST | 0.026 | <0.001 |  | AST | -0.061 | 0.023 |
|  | Cancer | 0.043 | 0.027 |  | Cancer | 0.048 | 0.078 |
|  | Smoke | 0.003 | 0.019 |  | Smoke | 0.020 | 0.663 |
|  | Thyroid problem | -0.017 | 0.038 |  | Thyroid problem | -0.007 | 0.001 |
|  | Gout | 0.030 | <0.001 |  | Gout | -0.084 | 0.029 |
|  | Diebetes | 0.002 | 0.028 |  | Diebetes | 0.039 | 0.038 |
|  | Hypertension | -0.029 | <0.001 |  | Hypertension | -0.004 | <0.001 |
|  | Stroke | 0.015 | 0.009 |  | Stroke | 0.070 | 0.032 |
|  | Arthritis | -0.047 | 0.017 |  | Arthritis | 0.059 | <0.001 |
|  | High cholesterol | 0.078 | 0.022 |  | High cholesterol | -0.023 | 0.591 |

SII: systemic immune-inflammatory index; SIRI: systemic inflammatory response index

Table S3 Threshold effect analysis of SII and SIRI on the CHD prevalence in the asthmatic population using the two-piece wise linear regression model

| SII |  |
| --- | --- |
| Fitting by the standard linear model | 0.026 (0.004, 0.048) |
| Fitting by the two-piece wise linear model | 0.0308 |
| Inflection point | 411.238 |
| SII < 411.238 (1000 cell/uL) | 0.014 ( 0.007, 0.021) |
|  | < 0.001 |
| SII > 411.238 (1000 cell/uL) | 0.031 (0.018, 0.044) |
|  | < 0.001 |
| Log likelihood ratio | < 0.001 |
| SIRI |  |
| Fitting by the standard linear model | 0.037 (0.001, 0.073) |
| Fitting by the two-piece wise linear model | 0.0251 |
| Inflection point | 1.812 |
| SII < 1.812 (1000 cell/uL) | 0.023 ( 0.002, 0.044) |
|  | < 0.001 |
| SII > 1.812 (1000 cell/uL) | 0.027 (0.006, 0.048) |
|  | < 0.001 |
| Log likelihood ratio | 0.031 |

SII: systemic immune-inflammatory index; SIRI: systemic inflammatory response index
